# Hybrid Plasmids Encoding Antimicrobial Resistance and Virulence Traits Among Hypervirulent *Klebsiella pneumoniae* ST2096 in India

**DOI:** 10.3389/fcimb.2022.875116

**Published:** 2022-04-27

**Authors:** Chaitra Shankar, Karthick Vasudevan, Jobin John Jacob, Stephen Baker, Barney J. Isaac, Ayyan Raj Neeravi, Dhiviya Prabaa Muthuirulandi Sethuvel, Biju George, Balaji Veeraraghavan

**Affiliations:** ^1^ Department of Clinical Microbiology, Christian Medical College and Hospital, Vellore, India; ^2^ Cambridge Institute of Therapeutic Immunology & Infectious Disease (CITIID), Department of Medicine, University of Cambridge, Cambridge, United Kingdom; ^3^ Department of Pulmonary Medicine, Christian Medical College and Hospital, Vellore, India; ^4^ Department of Haematology, Christian Medical College and Hospital, Vellore, India

**Keywords:** hypervirulent, *K. pneumoniae*, ST2096, hybrid plasmid, CRISPR-Cas, multidrug resistance

## Abstract

**Background:**

Hypervirulent variants of *Klebsiella pneumoniae* (HvKp) were typically associated with a broadly antimicrobial susceptible clone of sequence type (ST) 23 at the time of its emergence. Concerningly, HvKp is now also emerging within multidrug-resistant (MDR) clones, including ST11, ST15, and ST147. MDR-HvKp either carry both the virulence and resistance plasmids or carry a large hybrid plasmid coding for both virulence and resistance determinants. Here, we aimed to genetically characterize a collection of MDR-HvKp ST2096 isolates haboring hybrid plasmids carrying both antimicrobial resistance (AMR) and virulence genes.

**Methods:**

Nine *K. pneumoniae* ST2096 isolated over 1 year from the blood sample of hospitalized patients in southern India that were MDR and suspected to be HvKp were selected. All nine isolates were subjected to short-read whole-genome sequencing; a subset (n = 4) was additionally subjected to long-read sequencing to obtain complete genomes for characterization. Mucoviscosity assay was also performed for phenotypic assessment.

**Results:**

Among the nine isolates, seven were carbapenem-resistant, two of which carried *bla*_NDM-5_ on an IncFII plasmid and five carried *bla*_OXA-232_ on a ColKP3 plasmid. The organisms were confirmed as HvKp, with characteristic virulence genes (*rmpA2*, *iutA*, and *iucABCD*) carried on a large (~320 kbp) IncFIB–IncHI1B co-integrate. This hybrid plasmid also carried the *aadA2*, *armA*, *bla*_OXA-1_, *msrE*, *mphE*, *sul1*, and *dfrA14* AMR genes in addition to the heavy-metal resistance genes. The hybrid plasmid showed about 60% similarity to the IncHI1B virulence plasmid of *K. pneumoniae* SGH10 and ~70% sequence identity with the first identified IncHI1B pNDM-MAR plasmid. Notably, the hybrid plasmid carried its type IV-A3 CRISPR-Cas system which harbored spacer regions against *traL* of IncF plasmids, thereby preventing their acquisition.

**Conclusion:**

The convergence of virulence and AMR is clinically concerning in *K. pneumoniae*. Our data highlight the role of hybrid plasmids carrying both AMR and virulence genes in *K. pneumoniae* ST2096, suggesting that MDR-HvKp is not confined to selected clones; we highlight the continued emergence of such genotypes across the species. The convergence is occurring globally amidst several clones and is of great concern to public health.

## Introduction

*Klebsiella pneumoniae* (Kp) is a common cause of hospital-acquired infection (Marr and Russo, 2019). Some forms of *K. pneumoniae* can cause invasive diseases, affecting the liver and other internal organs, and are considered to be hypervirulent (HvKp) (Shon et al., 2013; Marr and Russo, 2019). While HvKp does not have a precise definition, it refers to isolates that carry the virulence plasmid (GenBank accession numbers CP025081, AY378100) coding for *rmpA/rmpA2*, *iucA*, *iutA*, and/or *iroB* (Marr and Russo, 2019). Although HvKp was confined to community-acquired infections, recent reports suggest that HvKp is an emerging nosocomial pathogen with the potential to cause devastating hospital outbreaks thereby establishing itself in both niches (Gu et al., 2018; Liu et al., 2020). HvKp infections are becoming prevalent globally and associated with increased mortality, and a recent study reports the gut colonization of MDR-HvKp in pregnant women (Shon et al., 2013; Marr and Russo, 2019; Huynh et al., 2020). Earlier, HvKp isolates were susceptible to the majority of clinically relevant antimicrobials as they were rarely associated with multidrug resistance plasmids (Shon et al., 2013). However, in the last decade, the organism has undergone several genomic changes and expanded its genome by acquiring multiple resistance plasmids (Lee et al., 2017; Gu et al., 2018; Liu et al., 2020).

It has been found that in the population structure of HvKp, when determined by multi-locus sequence typing (MLST) and whole-genome sequencing (WGS), most HvKp isolates belong to the clonal groups (CG) 23, 65, 86, 375, and 380 (Bialek-Davenet et al., 2014). Conversely, carbapenem-resistant *K. pneumoniae* (CRKp) is associated with a clonal expansion of CG258 in Europe and endemic dissemination of ST11, ST14, ST147, and ST231 clones in Asia and Europe (Qi et al., 2011; Lee et al., 2016; Navon-Venezia et al., 2017; Wyres et al., 2020). However, there have been recent reports on the the emergence of HvKp with multidrug resistance (MDR, resistant to one or more agents in ≥3 antimicrobial classes) phenotypes in divergent CGs (Lee et al., 2017; Turton et al., 2018; Lam et al., 2019), creating new strains with the ability to cause serious infection with limited treatment options (Bialek-Davenet et al., 2014; Yao et al., 2018; Yang et al., 2020). The convergence of MDR and virulence pathotypes in a single isolate occurs either by the uptake of a virulence plasmid by MDR isolate or by the uptake of plasmids carrying antimicrobial resistance genes (ARGs) by the virulent isolates (Tang et al., 2020). CRKp ST11 acquiring the pLVPK-like virulence plasmid and ST23 HvKp acquiring multiple resistance plasmids are some instances where both pathotypes have converged (Liu et al., 2020; Shankar et al., 2020).

Specifically, carbapenem-resistant hypervirulent *K*. *pneumoniae* (CR-HvKp) has arisen by the formation of mosaic plasmids and hybrid plasmids (Tang et al., 2020; Yang et al., 2021). These mosaic plasmids are typically composed of two or more different plasmid backbones and create a scenario where AMR and virulence determinants are encoded on a single large plasmid with a mosaic (medley) arrangement of resistance and virulence genes whereas the hybrid plasmids are co-integrates with two plasmid backbones (Lam et al., 2019; Turton et al., 2019). Mosaic plasmids with fragments of virulence plasmid and IncFII_K_ coding for resistance and virulence have been described among ST15 *K. pneumoniae* from Europe (Lam et al., 2019). In contrast, the hybrid plasmids with a range of replicons (IncFIB–IncHI1B, IncFIBK–IncHI1B, IncFIB–IncR) have been described in China and Europe (Lam et al., 2019; Turton et al., 2019; Li et al., 2020; Xie et al., 2020; Yang et al., 2020) and are being increasingly reported among regional MDR clones. Our understanding of these CR-HvKp hybrid plasmids is limited due to an insufficient number of complete plasmid sequences. Here, we aimed to characterize a set of MDR-HvKp belonging to ST2096, possessing hybrid plasmids that simultaneously carry both AMR and virulence genes. The complete genome sequences of four of these isolates were further generated by long-read sequencing to elucidate the detailed structure of the hybrid plasmid *via* comparative genomics.

## Materials and Methods

### Bacterial Isolates

The *K. pneumoniae* were isolated from patients with bacteremia admitted to the Christian Medical College, Vellore, India, in 2019. The isolates were identified using standard microbiological methods and further confirmed by VITEK MS [Database v2.0, bioMerieux, Marcy-l’Étoile, France] (Versalovic et al., 2011). The isolates were screened for hypermucoviscous phenotype using the string test (Shon et al., 2013). In addition, the mucoid phenotype-associated genes *rmpA* and *rmpA2* were detected by PCR (Turton et al., 2010; Compain et al., 2014). The demographic and clinical details of the nine patients from whom the organisms were isolated were accessed from electronic medical records. The study was approved by the Institutional Review Board of Christian Medical College, Vellore, with minute number 9616 (01/09/2015).

### Antimicrobial Susceptibility Testing

Antimicrobial susceptibility testing (AST) was performed using the Kirby-Bauer disk diffusion method according to the CLSI 2019 guidelines (Clinical and Laboratory Standards Institute, 2019). The tested antimicrobials were cefotaxime (30 µg), ceftazidime (30 µg), piperacillin/tazobactam (100/10 µg), cefoperazone/sulbactam (75/30 µg), imipenem (10 µg), meropenem (10 µg), ciprofloxacin (5 µg), levofloxacin (5 µg), gentamicin (10 µg), amikacin (30 µg), and minocycline (30 µg). The minimum inhibitory concentration (MIC) of meropenem was determined by the broth microdilution (BMD) method. *Escherichia coli* ATCC^®^ 25922, *Enterococcus faecium* ATCC^®^ 29212, and *Pseudomonas aeruginosa* ATCC^®^ 27853 were used as controls. Data were interpreted according to the 2019 CLSI guidelines (CLSI 2019).

### Mucoviscosity Assay

Overnight culture of the HvKp isolates was inoculated in Luria Bertani (LB) broth (Oxoid, Hampshire, United Kingdom) and centrifuged at 1,000 rpm for 15 min as previously described (Mike et al., 2021). Briefly, the optical density (OD) of the supernatant was measured at 600-nm wavelength in the UV spectrophotometer (1st OD). 1 ml of PBS (phosphate-buffered saline) was added, and OD_600_ was adjusted to 1.00. This was centrifuged again at 1,000 rpm for 5 min, and the OD_600_ of the supernatant (3rd OD) was measured. Non-virulent isolate K. quasipneumoniae ATCC® 700603 was used as a control for mucoviscosity assay. This assay is based on the principle that hypermucoviscous isolates do not sediment easily and hence the OD_600_ after centrifugation will be higher than the counterparts of control and non-hypermucoviscous isolates. Hence, hypermucoviscous isolates will have a higher SAC ratio than the rest.

Sedimentation Assay Calculation (SAC) = Reading of 3rd OD_600_/Reading of 1st OD_600_.

### DNA Extraction and Genome Sequencing

The isolates studied were revived from the archive of the Department of Clinical Microbiology, and a single colony was inoculated in LB broth at 37°C. Total genomic DNA was extracted from pelleted cells using the Wizard DNA Purification Kit (Promega, Madison, WI, USA). Extracted DNA was quantified using NanoDrop One spectrophotometry (Thermo Fisher Scientific, Waltham, MA, USA) and Qubit 3.0 fluorometry (Life Technologies, Carlsbad, CA, USA) and stored at -20°C until further use.

A sequencing library was prepared using the Nextera DNA Flex Library Preparation Kit (Illumina, San Diego, CA, USA). Subsequently, the paired-end library was subjected to sequencing on a HiSeq 2500 platform (Illumina, USA) generating 2 × 150-bp reads. Sequencing reads with a PHRED quality score below 20 were discarded, and adapters were trimmed using cutadapt v1.8.1 and assessed with FastQC v0.11.4 (Andrews, 2010; Martin, 2011). For a subset of four isolates, long-read sequencing was performed using an Oxford Nanopore MinION FLO-MIN106 R9 flow cell (Oxford Nanopore Technologies, UK). The long-read DNA library was prepared using the SQK-LSK108 ligation sequencing kit (v.R9) along with the ONT EXP-NBD103 Native Barcode Expansion kit (Oxford Nanopore Technologies, Oxford, UK). The library was loaded onto flow cells, run for 48 h using the standard MinKNOW software (Guppy version 3.6).

### Genome Assembly and Evaluation

Draft genome sequence data generated using Illumina were assembled using SPAdes (v.3.13.0) (Bankevich et al., 2012). A hybrid *de novo* assembly was generated for a subset of four isolates (Vasudevan et al., 2020). The nanopore long reads were error-corrected with the standalone Canu error correction tool (v.1.7) and assembled using the Unicycler hybrid assembly pipeline (v 0.4.6) with the default settings (Koren et al., 2017; Wick et al., 2017). The genome sequences were polished using high-quality Illumina reads, as described previously using Pilon (Walker et al., 2014). The assembled genomes were subjected to quality assessment using CheckM v1.0.5 (Parks et al., 2015) and Quast v4.5 (Gurevich et al., 2013). *K. pneumoniae* NTUH-K2044 (GenBank accession number AP006725) was used as the reference genome since it is a well-characterized type of strain of ST23 hypervirulent *K. pneumoniae*.

### Genome Analysis

Genome assemblies were submitted to NCBI GenBank and annotated using the NCBI Prokaryotic Genome Annotation Pipeline [PGAP v.4.1] (Tatusova et al., 2016).

The genomes described in the study are publicly available under the Bioproject ID PRJNA613369 in GenBank with accession numbers CP053765–CP053770, CP053771–CP053780, CP058798–CP058806, JAARNO010000001.1–JAARNO010000005.1, JAAQSG000000000, JAARNJ000000000, JAARMH000000000, and JAAQTC000000000. The antimicrobial resistance profile of the assembled genome sequences was identified using ResFinder v.4.1 available from CGE server (Bortolaia et al., 2020). Similarly, the presence of plasmids in the genomes was identified and characterized using PlasmidFinder (v.1.3) available at the CGE server (Carattoli et al., 2014). MLST and virulence loci (yersiniabactin, aerobactin, and other siderophore production systems) were identified using Kleborate (v.2.0.0) (Lam et al., 2021). The presence of virulence factors was confirmed using the virulence database at Pasteur Institute for *K. pneumoniae* (Jolley and Maiden, 2010). YbST and AbST, typing schemes based on yersiniabactin and aerobactin loci, were deduced from the database at Pasteur Institute for *K. pneumoniae* (https://bigsdb.pasteur.fr/cgi-bin/bigsdb/bigsdb.pl?db=pubmlst_klebsiella_seqdef&page=profiles). The K and O antigen loci were identified using Kaptive available at Kleborate (Wyres et al., 2016; Wick et al., 2018). The final assembled circular chromosomes and plasmids were visualized using CGView server v.1.0 (Grant and Stothard, 2008) and Easyfig (Sullivan et al., 2011). CRISPR regions in the genomes were identified with the CRISPRCasTyper web server (Russel et al., 2020). The genetic distance between isolates was calculated using average nucleotide identity (ANI) available at OrthoANI (Lee et al., 2016). Pairwise distance between the nine isolates was determined with BA10835 as reference using SNP-dists v 0.6.3 (Wysocka et al., 2020) from the raw reads by aligning the short reads of each isolate against the reference. An SNP-based phylogenetic tree of the complete hybrid plasmids with IncHI1B–IncFIB (pNDM-MAR) replicon types which are mentioned in **Table S1** was constructed using CSI phylogeny (https://cge.cbs.dtu.dk/services/CSIPhylogeny/).

## Results

### Clinical Manifestations and Microbiological Characteristics of the Isolates

During the routine surveillance of HvKp, we identified isolates that were negative for the string test, positive for *rmpA2* as determined by PCR, and carbapenem-resistant as determined by AST. These isolates were chosen for whole genome sequencing, and we identified nine ST2096 (a single-locus variant of ST14) *K. pneumoniae* associated with bacteremia in our hospital (**Table 1**). These nine *K. pneumoniae* ST2096 were resistant to all tested antimicrobials by disk diffusion assay and were initially considered to be extensively drug-resistant (XDR, non-susceptible to at least one agent in all but ≤2 classes of antimicrobials). However, upon MIC testing, two isolates were found to be susceptible to meropenem (MIC ≤0.5 µg/ml). The results of the mucoviscosity assay are mentioned in **Figure S1**. The isolates showed significantly higher OD_600_ when compared to the control strain indicating the lack of sedimentation by the hypervirulent isolates.

**Table 1:** The demographic and clinical details of the patients with bacteremia caused by hypervirulent *K. pneumoniae* ST2096.

Micro no.	Month of isolation	Unit	Clinical manifestation	Risk factors	Prior hospitalization	Therapy administered and duration of therapy		Outcome
BA1602	January 2019	Surgery	Carcinoma ascending colon	Anastomotic leak with fecal peritonitis, MODS, fever, Cough	Yes	Polymyxin B, meropenem, teicoplanin, tigecycline	16 days	Succumbed to death
BP3636	March 2019	Hematology	Fever and giddiness	Congenital sideroblastic anemia, Stem cell transplant—day 28	Yes	Meropenem, tigecycline, fosfomycin, colistin	2 days	Succumbed to death
BA10334	April 2019	Gastroenterology	Persistent rise of temperature, recurrent vomiting, loss of weight	Disseminated tuberculosis, sepsis, pleural effusion	Yes, treated elsewhere for 10 days	Cefoperazone-sulbactam, meropenem, colistin, vancomycin	10 days	Succumbed to death
BA10835	April 2019	Hepatology	Acute febrile illness with Jaundice	Acute on chronic liver failure, portal hypertension, Wilson disease	No	Tigecycline	1 month	Recovered
BA25425	August 2019	Neurosurgery	Road traffic accident—head injury	Right subdural hematoma Temporal hemorrhagic contusion	Yes	Linezolid, piperacillin-tazobactam, cefoperazone-sulbactam, gentamicin	1 month	Succumbed to death
BA27935	September 2019	Casualty	Acute febrile illness with altered sensorium	Hypertension, intracranial bleed Hemiplegia	Yes, treated elsewhere for 10 days	Meropenem	1 day	Discharged against medical advice
BA28118	September 2019	Hematology	Acute promyelocytic leukemia Acute kidney injury	Fever, multiple episodes of bleeding from gums/per rectum	No	Meropenem, tigecycline, polymyxin B, amikacin	1 month	Recovered
BA32040	October 2019	Hematology	Acute febrile illness	Beta thalassemia post-allogenic stem cell transplant—day 280 Skin GVHD	Yes	Colistin, meropenem	15 days	Recovered
BA39100	December 2019	Hematology	Extramedullary granulocytic sarcoma	Invasive mucormycosis	Yes	Meropenem, tigecycline Teicoplanin polymyxin B	3 days	Recovered

GVHD, graft versus host disease; MODS, multiple-organ dysfunction syndrome.

From the resulting nine genome sequences, the surface capsule (K) loci were predicted to be K64 whereas the O-antigen encoding loci was determined to be O1v1 in all isolates (**Tables 2** and **3**). The pairwise average nucleotide identity (ANI) among the nine draft genomes was >99.8% (**Figure S2A**). The pairwise SNP difference among the nine isolates segregated them into two clusters, with BA10835 and BA27935 being >260 SNPs from the remaining seven sequences (**Figure S2B**). Within the major cluster (7 isolates), BA10334 and BA1602 were highly related (2 SNPs), and similarly, BA25425 and BP3636 (6SNPs) were related. Since this is a retrospective study, other specimen sources were not investigated to determine if there was an outbreak of ST2096 in the hospital and hence the dissemination of the plasmid cannot be explained.

**Table 2:** Phenotypic and genotypic characteristics obtained using hybrid genome assembly of four Indian MDR hypervirulent *K. pneumoniae* ST2096.

Isolate ID	BA10835	BA27935	BP3636	BA32040
Accession numbers	CP053765–CP053770	CP058798–CP058806	CP053771–CP053780	JAARNO010000001.1 to JAARNO010000005.1
Meropenem MIC	128 µg/ml	4 µg/ml	64 µg/ml	≤0.5µg/ml
Chromosomal AMR genes	*aac(6′)-lb-cr*, *bla*_SHV_, *bla*_OXA-1_, *fosA, dfrA1*	*aac(6’)-Ib-cr, bla*_SHV_, *bla*_OXA-1_, *fosA*, *dfrA1*	*bla*_SHV_, *fosA, dfrA1*	*aac(6’)-Ib-cr*, *bla*_SHV_, *bla*_OXA-1_, *fosA, dfrA1*
Chromosomal virulence genes	*fyuA, irp1, kfuABC, mrkACFJ, ybtAEPQSTUX*	*fyuA, irp1, kfuABC, mrkACFJ, ybtAEPQSTUX*	*fyuA, irp1, irp2, kfuABC, mrkABCDFHIJ, ybtAEPQSTUX*	*fyuA, irp1, kfuABC, mrkABCDFHIJ, ybtAEPQSTUX*
No. of plasmids	5	4	4	4
IncHI1B/IncFIB (pNDM-MAR) **Virulence plasmid**	*aadA2, armA*, *bla*_TEM-1B_, *bla*_CTX-M-15_, *mphE, msrE, sul1, tetD, dfrA12*	*aadA2, armA*, *bla*_TEM-1B_, *bla*_CTX-M-15_ *, mphE, msrE*, *sul1, tetD, dfrA12*	*aadA2, armA*, *aac(6’)-Ib-cr, bla*_TEM-1A_, *bla*_CTX-M-15_, *bla*_OXA-1_, *mphE, msrE, sul1, tetD, dfrA12, dfrA14*	*bla*_TEM-1A_, *bla*_CTX-M-15_, *tetD, dfrA14*
	iucABCD, iutA, rmpA2*	iucABCD, iutA, rmpA2*	iucABCD, iutA, rmpA2*	iucABCD, iutA, rmpA2*
IncFIBK-IncFIIK	*catA1*	absent	No AMR gene	No AMR gene
ColKP3	absent	absent	*bla*_OXA-232_	absent
IncFII	*aadA2, rmtB*, *bla*_NDM-5_, *ermB, mphA, sul1, dfrA12*	*aadA2, rmtB*, *bla*_NDM-5_, *bla*_TEM-1B_ *, ermB, mphA, sul1, dfrA12*	Absent	*rmtB*, *bla*_TEM-1B_, *ermB, mphA*
Other plasmids	ColRNAI	Col(BS512), ColRNAI	ColRNAI	ColRNAI

rmpA2*, rmpA2 allele number 8 was observed which is frameshifted; MIC, minimum inhibitory concentration determined by broth microdilution; AMR, antimicrobial resistance.

**Table 3:** Genotypic characteristics of multidrug-resistant hypervirulent *K. pneumoniae* belonging to ST2096 obtained from short read assembly.

Accession number and isolate ID	*rmpA* and/or *rmpA2*	Capsule type	O antigen	Ybt, ICEKp	Resistance genes	Plasmids	Virulence genes
JAARMH000000000 BA1602	rmpA2*	K64	O1v1	*ybt14*; ICEKp5	*aac(6)-Ib-cr, aadA2, armA, bla*_SHV-106_ *, bla*_CTX-M-15_ *,bla*_OXA-1_ *, bla*_TEM-150_ *, fosA6, mphE, msrE, sul1, tetD, dfrA1, dfrA12, dfrA14*	ColKP3, IncFIBK, incFIB (pNDM-MAR), IncHI1B (pNDM-MAR)	*fyuA, irp1, irp2, kfuAB*, aerobactin, *mrkABCDFHIJ*
JAAQTC000000000 BA25425	rmpA2*	K64	O1v1	*ybt14*; ICEKp5	*aadA2, armA, sat-2A, bla*_SHV-28_ *, bla*_CTX-M-15_ *,bla*_OXA-1_ *, bla*_TEM-1D_ *, bla*_OXA-232_ *, mphE, msrE, sul1, tetD, dfrA1, dfrA12, dfrA14*	ColKP3, ColRNAI, IncFIBK, IncFIB (pNDM-Mar), IncHI1B (pNDM-MAR)	*fyuA, irp1, irp2, kfuABC, mrkABCDFHIJ*
JAAQSG000000000 BA28118	rmpA2*	K64	O1v1	*ybt14*; ICEKp5	*aadA2, armA, bla*_SHV-28_ *, bla*_CTX-M-15_ *, bla*_OXA-1_ *, bla*_TEM-1D_ *, bla*_OXA-232_ *,mphE, msrE, sul1, tetD, dfrA12, dfrA14*	IncFIBK, IncFIB(pNDM-Mar), ColKP3, ColBS512, IncHI1B (pNDM-MAR)	*fyuA, irp1, irp2*, aerobactin, *kfuABC, mrkABCDFHIJ*
JAARNJ000000000 BA39100	*rmpA2*	K64	O1v1	*ybt14*; ICEKp5	*aac(6)-lb-cr, aadA2, armA, sat2A, bla*_SHV-106_ *, bla*_CTX-M-15_ *, bla*_OXA-1_ *, bla*_TEM-1B_ *, bla*_OXA-232_ *, catB, fosA6, mphE, msrE, sul1, tetD, dfrA1, dfrA12, dfrA14*,	ColKP3, IncFIBK, IncFIB(pNDM-Mar), IncHI1B (pNDM-MAR)	*fyuA, irp1, irp2, kfuA, kfuC*, aerobactin, *mrkABCDFHIJ*
JAAQSS00000000 BA10334	rmpA2*	K64	O1v1	*ybt14*; ICEKp5	*aac(6)-lb-cr, aadA2, armA, bla*_SHV_ *, bla*_CTX-M-15_ *, bla*_OXA-1_ *, bla*_TEM-1A_ *, bla*_OXA-232_ *, fosA, msrE, mphE, sul1, tetD, dfrA1, dfrA12, dfrA14*	ColKP3, IncHI1B (pNDM-MAR), IncFIB (pNDM_MAR), IncFIBK	*fyuA, irp1, irp2,iutA*, aerobactin, *kfuABC, mrkABCDFHIJ*

rmpA2*, frameshift mutation.

### Characterization of the HvKp ST2096 Chromosomes

**Figure 1:**
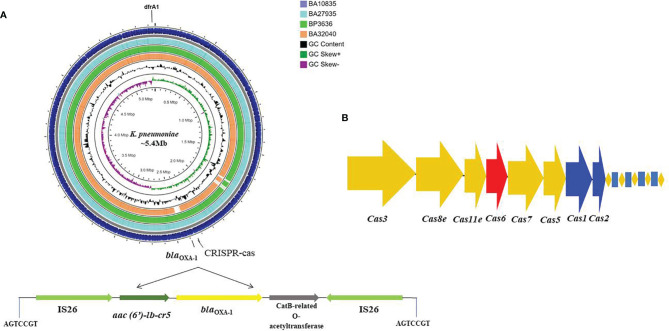
Circular genome map of four MDR hypervirulent *K. pneumoniae* ST2096 chromosomes. **(A)** Circles from the outside to the inside show the CDS region of BA10835 (blue), BA27935 (cyan), BP3636 (green), BA32040 (yellow), GC skew (dark green and purple), and GC content (black). Linear view of the IS*26* mediated trans locatable units carrying *aac(6′)-Ib-cr* (fluoroquinolones and aminoglycosides), *bla*_OXA–1_ (ampicillin), and *catB3* (chloramphenicol) inserted to the chromosome. A repeat region of 7 bases read as AGTCCGT was present on either ends where the insertion was observed. Map generated using CGView server (Grant and Stothard, 2008). **(B)** Type I-E CRISPR-Cas system identified in the chromosomes with repeat region of 28 bases.

Typically, *K. pneumoniae* chromosomes are characterized by the presence of *bla*_SHV_ and *fosA*. In addition to these resistance genes, surprisingly, we found that three of the four MDR-HvKp isolates with complete genomes had the *aac(6′)-lb-cr*, *bla*_OXA-1_ and *dfrA1* genes integrated into chromosome on mobile genetic elements. Specifically, *aac(6′)-lb-cr* and *bla*_OXA-1_ were inserted by IS*26* in the central region of the chromosome at ~2.3 Mbp with a 7-bp flanking region (AGTCCGT) (**Figure 1A**). The *dfrA1* gene was associated with IS*Kpn26* and a class 1 integron, *intI1*, at position ~5.3 Mbp. From the nine draft genome sequences, we identified a type I-E CRISPR located on the chromosome, characterized by 7–12 spacers of 32 bp and an adjacent IS*Kpn26* (**Figure 1B**).

The key virulence determinant carried by the chromosome of *K. pneumoniae* is the *ybt* locus, which is mobilized by ICEKp. *ybt14* was carried on ICEKp5 and integrated into the chromosome in all nine sequenced isolates. The *fyuA* and *irp1* yersiniabactin receptors were also present on the chromosome, along with a *kfu* gene cluster encoding for iron uptake and the *mrk* gene cluster, which facilities biofilm formation. YbST, typing based on yersiniabactin loci, classified all the isolates as belonging to YbST140.

The genomes of BP3636 (5,352,701 bp) and BA32040 (5,298,155 bp) were smaller than the genomes of BA10835 (5,355,460 bp) and BA27935 (5,356,693 bp) and lacked some of the iron transporters and metal transporter-encoding genes on the chromosomes. In addition, BA32040 lacked some of the genes coding for ABC transporter, MFS transporter, and LysE and LysR family transcriptional regulators when compared to the other three complete genomes (data not shown).

### Characterization of the Plasmids Among HvKp ST2096

**Figure 2:**
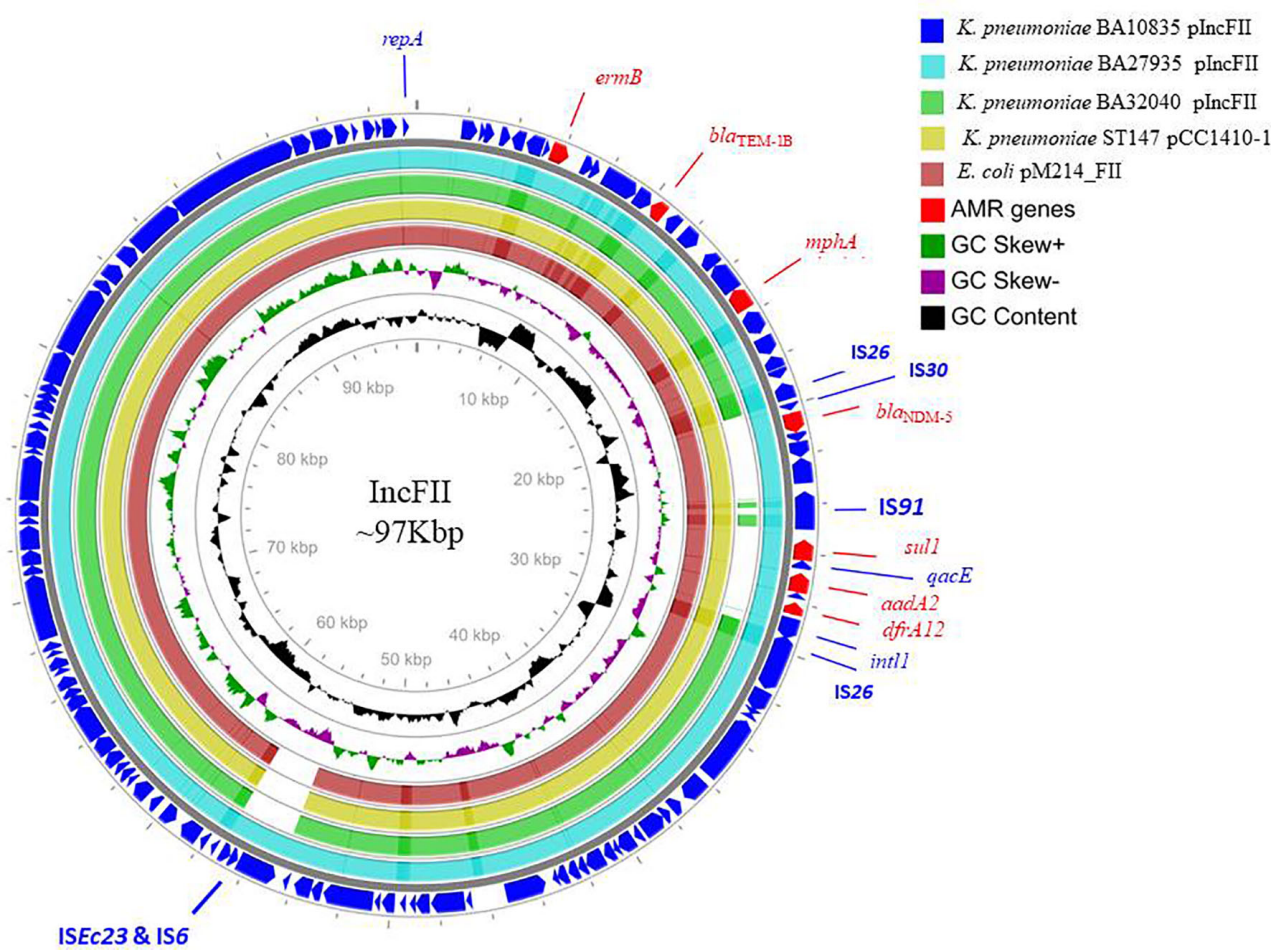
Alignment of IncFII plasmids of three MDR hypervirulent *K. pneumoniae* belonging to ST2096. Circles from the outside to the inside show the CDS region of BA10835 (blue), BA27935 (cyan), BA32040 (green), and nearest matching reference plasmids that belong to *K. pneumoniae* pCC1410-1 (yellow; KT725788) and *E. coli* pM214 (red; AP018144). GC skew (dark green and magenta) and GC content (black) of the plasmid are represented in the inner circles. Maps were generated using the CGView server.

The nine HvKp isolates were found to possess an array of AMR genes associated with 4–5 plasmids per genome, including the virulence plasmid (**Tables 2** and **3**). Notably, *bla*_NDM-5_ was carried on the IncFII plasmid (~97 kbp) along with *aadA2*, *rmtB*, *ermB*, *mphA*, *sul1*, *dfrA12*, and *bla*_TEM-1B_ (**Figure 2**). We also found a 293-bp segment of an IS30 family transposase, with similarity to the IS*Aba125*, adjacent to *bla*_NDM-5_. The closest matching plasmid from the global database was from *K. pneumoniae* JUNP055 (GenBank accession no. LC506718), which also harbored *bla*_NDM-5_ but lacked a few IS elements (IS*Ec23* and IS6 family) when compared to IncFII of the ST2096 isolates (**Figure 2**). These pJUNP055 and IncFII plasmids from the present study shared ~80% sequence identity to those of *E. coli* M105 from Myanmar (GenBank accession no. AP018136), which lacked *bla*_NDM-5_. As predicted, the *bla*_OXA-232_ carbapenemase was encoded by a small 6Kb ColKP3 plasmid and was adjacent to a truncated IS*Ecp1* (207 bp). Notably, the two isolates (BA32040 and BA1602) that were susceptible to meropenem lacked a carbapenemase-encoding gene. Additionally, a large (~307-kbp) plasmid was present in all four of the assembled genomes and was found to be a fusion of IncFIB and IncHI1B plasmid backbones, carrying both AMR genes and virulence genes which will be referred to as p2096_hyb (**Table 2**). The isolates also harbored several small plasmids (<8 kbp), such as ColRNAI and Col(BS512), which did not encode either AMR or virulence genes.

### Hybrid Plasmid Coding for Virulence and Antimicrobial Resistance

A large hybrid virulence plasmid, p2096_hyb, of ~307 kbp was the hallmark of all ST2096 isolates, and they carried a frameshifted *rmpA2* and the aerobactin siderophore, encoded by *iucABCD*. The hybrid plasmid in the four isolates BA10835, BA27935, BP3636, and BA32040 will be referred to as p10835_hyb, p27935_hyb, p3636_hyb, and p32040_hyb, respectively. This plasmid carried both IncHI1B and IncFIB replicons on the pNDM-MAR backbone and hence was called a hybrid plasmid. This plasmid-encoded several AMR genes as listed in **Table 2**. The backbone of the plasmid consisted of genes related to replication, toxin–antitoxin system, conjugative transfer, DDE transposase, transcriptional regulators, and tyrosine-specific recombinases. Notably, *dfrA12*, *aadA2*, and *sul1* genes were inserted into the virulence plasmid through a class 1 integron, *intI1*. The insertion of *bla*_OXA-1_, *catB*, and *aac(6′)-lb-cr5* on the hybrid plasmid was through IS*26*, comparable to the arrangement observed in the chromosome (**Figures 1A, B**). A Tn*3* transposon contained several AMR genes including *msrE*, *mphE*, *sul1* and β-lactamases, such as *bla*_OXA-1_, *bla*_CTX-M-15_, and *bla*_TEM-1_. In addition to Tn*3*, *bla*_CTX-M-15_ and *bla*_TEM-1_ were associated with IS*Ec9*, a resolvase, and IS*91* insertion sequence. The hybrid plasmid in BA32040 was shorter (272 kbp) and lacked *aadA2*, *armA*, *bla*_OXA-1_, *msrE*, *mphE*, *sul*, *dfrA14* and the CRISPR array in comparison to plasmids found in the other three genomes.

**Figure 3:**
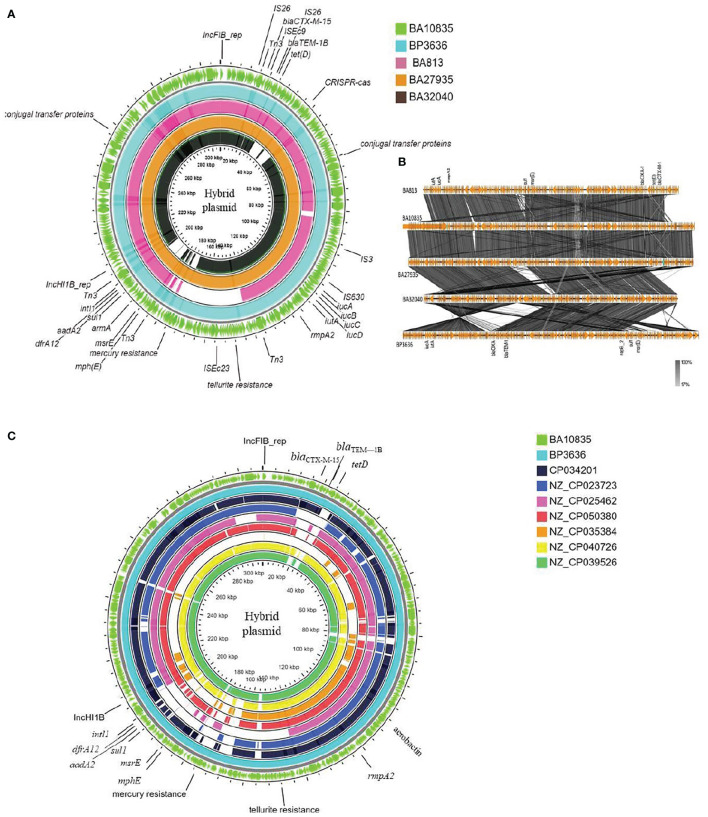
Maps of ST2096 mosaic plasmids in comparison to previously reported IncHI1B-IncFIB virulence plasmids. **(A)** Circular genome comparison map of IncFIB–IncHIB mosaic plasmids from outer to inner rings-BA10835 (light green), BP3636 (teal), BA813 (red), BA27935 (orange), and BA32040 (black). All the isolates belong to ST2096 and mosaic plasmid from BA813 (MK649825) was isolated during 2017 from the same study center. It lacks the heavy metal resistance encoding region when compared to other plasmids that were isolated during 2019. **(B)** Linear alignment of the mosaic plasmids obtained *K. pneumoniae* ST2096 using Easyfig. **(C)** Circular genome comparison map of IncHI1B–IncFIB mosaic plasmids from outer to inner rings-BA10835 (light green), BP3636 (teal), CP034201 (navy blue), NZ_CP023723 (indigo), NZ_CP025462 (pink), NZ_CP050380 (red), NZ_CP035384 (orange), NZ_CP040726 (yellow), and NZ_CP039526 (dark green). NZ_CP035384 shows the least similarity to the plasmids from the present study. Details of resistance and virulence genes carried by these plasmids are detailed in **Supplementary Table 1**.

Besides the virulence genes, p2096_hyb also carried genes encoding heavy metal tolerance such as *merARCTP* (mercury) and *terBEDWXZ* (tellurium) that were possibly inserted through the Tn*3* transposon, as shown in **Figure 3A**. Notably, a frameshift mutation was observed in *rmpA2* among all the isolates, which we presumed to be associated with the negative string test results as has been previously described (Yu et al., 2015; Shankar et al., 2021b). The frameshift occurred due to the deletion of an adenine base at the 346th base in *rmpA2*. Non-functional *rmpA2* and the absence of *rmpA* in these isolates contribute to the loss of a hypermucoid phenotype resulting from the decreased extracellular polysaccharide production. Aerobactin typing (AbST), a typing method using aerobactin alleles (*iucA*, *iucB*, *iucC*, *iucD*, and *iutA*), revealed all the study isolates that belonged to AbST-1. A type IV-A3 CRISPR-Cas system located on the hybrid plasmid of three isolates (BA10835, BA27935, BP3636) was characterized by the presence of 5–12 spacers and a 29-bp repeat region. One spacer each from the hybrid plasmid of the three isolates was comparable to *traL* of IncF plasmids that were found in *K. pneumoniae*, which may act as a potential obstacle in acquiring IncF plasmids and thereby limit the number of plasmids carried by these isolates.

**Figure 4:**
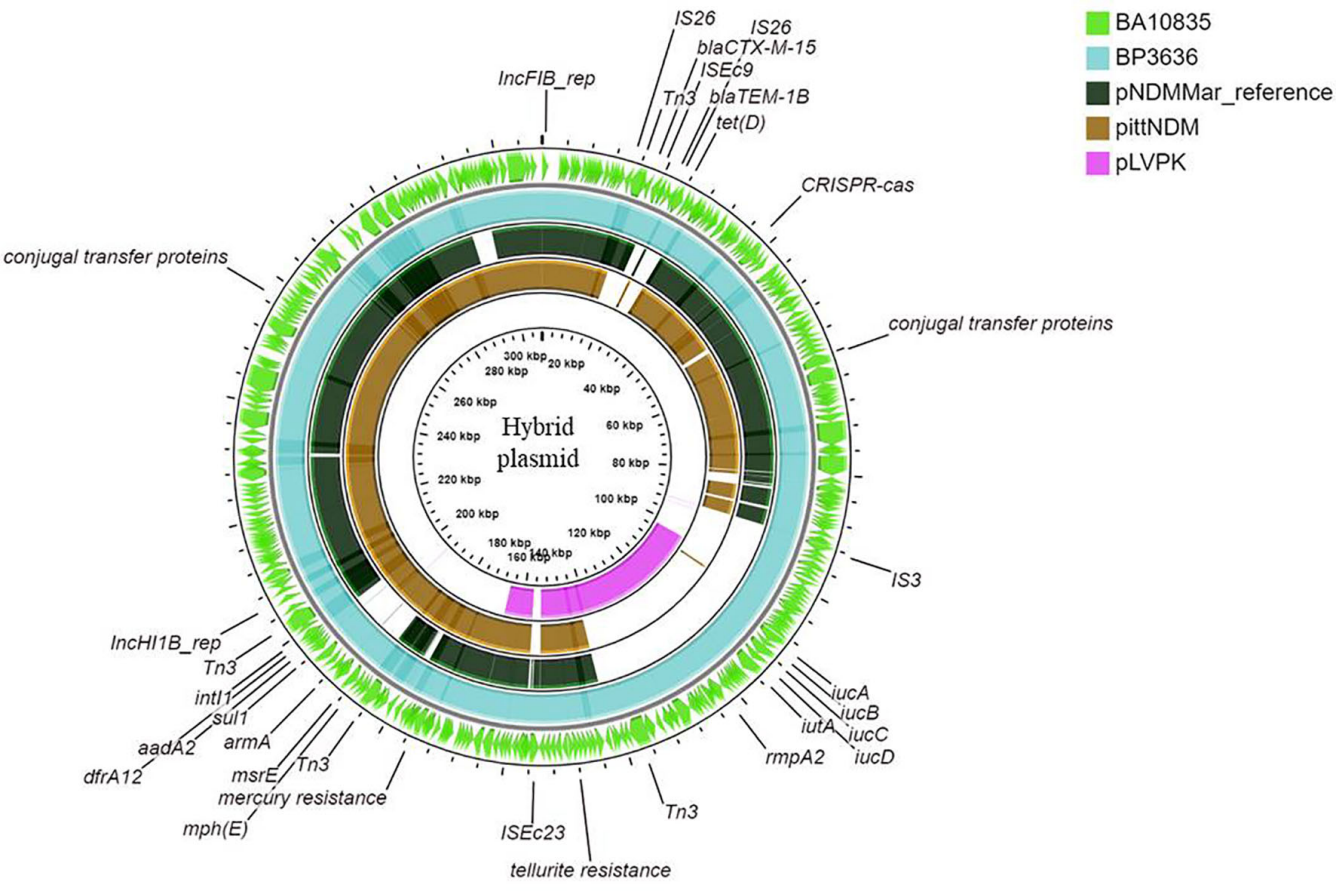
Maps of ST2096 mosaic plasmids in comparison to previously reported reference plasmids. Circular comparison map of plasmids from outer to inner rings—BA10836, BP3636, IncHI1B (pNDM-MAR, JN420336.1), PittNDM (NZ_CP006799.1), and pLVPK (AY378100.1) plasmids.

**Figures 3A** **, B** show the BLAST comparison of the hybrid plasmids from the present study to another hybrid plasmid, MK649825, from ST2096 isolated from the same center earlier in 2017 (Wyres et al., 2020). The plasmid, MK649825, was much smaller (273 kbp) than the plasmids isolated during 2019 and lacked the genes encoding mercury and tellurite tolerance. p2096_hyb of ~307 kbp showed ≤50% sequence identity with the pLVPK (Chen et al., 2004) reference virulence plasmid having in common with the region coding for virulence genes (**Figure S3**). p2096_hyb showed about 60% similarity to the IncHI1B virulence plasmid of *K. pneumoniae* SGH10 (Lam et al., 2018) and ~70% sequence identity with the first identified IncHI1B pNDM-MAR plasmid, a 267-kbp plasmid (GenBank accession no. JN420336.1) from a *K. pneumoniae* ST15 (Villa et al., 2012) carrying *bla*_NDM-1_ (**Figure S3**; **Figure 4**). While the p2096_hyb among HvKp ST2096 retained the regions coding for mercury, tellurite, β-lactam (*bla*_OXA-1_ and *bla*_CTX-M-15_), chloramphenicol, and aminoglycoside resistance, it had lost the segment carrying *bla*_NDM-1_ when compared to the pNDM-MAR plasmid. The insertion of the virulence-encoding region into the pNDM-MAR plasmid is possibly through the insertion mediated by IS*3* and IS*66* family proteins (IS*Ec23*) (**Figure 3A**). **Figure 4** shows the circular comparison of two-hybrid plasmids of ST2096 to pLVPK (GenBank accession no. AY378100.1), IncHI1B (pNDM-MAR) [GenBank accession no. JN420336.1], and pittNDM (GenBank accession no NZ_CP006799.1) plasmids. The latter two plasmids lack the virulence genes but encode antimicrobial resistance genes with IncHI1B backbone.

**Figure 3C** shows the BLAST comparison between the hybrid plasmid from the present study and the previously reported *K. pneumoniae* hybrid plasmids comprising IncHI1B (pNDM-MAR)–IncFIB (pNDM-MAR) replicon types. The complete sequences of these plasmids have been reported from 2015 till date among diverse clones of *K. pneumoniae* such as ST11, ST383, ST15, ST147, and ST23 and vary in size ranging from 261 to 396 kbp. The particulars of antimicrobial resistance and virulence genes carried by these plasmids are mentioned in **Table S1**. Interestingly, apart from the hybrid plasmid in the present study, another plasmid obtained in China, p44-1, from ST11 carried frameshifted *rmpA2*. Hybrid plasmids of HvKp ST2096, p10835_hyb (307 kbp), and p3636_hyb (317 kbp) showed high similarity to pKpvST383L (372 kbp) and pHB25-1-vir (396 kbp) which were isolated in England and China, respectively.

An SNP-based phylogenetic tree of the complete hybrid plasmids is shown in **Figure S4**. The tree was rooted at pAP855 since it was distant from the others and belonged to an ST23 isolate, lacking *rmpA2* (Tian et al., 2021). All the other plasmids belonged to MDR clones such as ST11, ST15, ST147, ST383, and ST2096. The plasmids from the present study formed a separate cluster coding for mercury and tellurite tolerance, truncated *rmpA2*, and lacking carbapenemase. This group is closely related to two plasmids from China isolated during 2015 and 2016, one of which also carries a truncated *rmpA2* (**Table S1**). The plasmids from England and Prague formed a separate group, and these carried *rmpA* and functional *rmpA2* and also encoded tellurite resistance determinants (**Table S1**).

## Discussion

MDR-Kp or CR-Kp isolates are clinically challenging, and specific carbapenemases are associated with regional/endemic clones in various regions (Lee et al., 2016; Shankar et al., 2021a). Recent reports describing the independent emergence of convergent HvKp isolates with hybrid and mosaic plasmids in multiple geographical locations make these organisms a major concern (Lam et al., 2019; Turton et al., 2019). Studies have described the acquisition of MDR plasmids by HvKp clones (ST23) and the acquisition of the pLVPK-like virulence plasmid by classical *K. pneumoniae* (cKp) causing invasive infections (Cejas et al., 2014; Chen and Kreiswirth, 2017; Xu et al., 2019; Shankar et al., 2020). This bidirectional convergence has resulted in the emergence of MDR-HvKp/CR-HvKp isolates within the nosocomial clones. Consequently, the circulation of nosocomial clones carrying a virulence plasmid is a matter of major public health concern (Zhan et al., 2017; Gu et al., 2018; Zhao et al., 2019; Liu et al., 2020). We report the acquisition of virulence factors among MDR clones, ST2096, crafting CR-HvKp in India.

In the present study, the ~307-kb hybrid plasmid was comparable to previously reported fusion plasmids including pKpvST147L [GenBank accession no. CM007852], pKpvST383L [GenBank accession no. CP034201], pKpvST147B [GenBank accession no. CP040726], and pBA813_1 GenBank accession no. [MK649825] (Turton et al., 2019; Wyres et al., 2020). Remarkably, these reference plasmids are associated with a diverse collection of clones, which were found to harbor *bla*_NDM_ and *bla*_OXA-48_ carbapenemase genes. Moreover, the insertion of resistance cassettes carrying *aadA2*, *armA*, *bla*_TEM-1B_, *bla*_CTX-M-15_, *mphE*, *msrE*, *sul1*, and *dfrA12* into hybrid plasmids of independent origin may accelerate the spread of MDR-HvKp. To date, the reports of hybrid plasmids are from clinical isolates indicating that the antimicrobial pressure present in this niche not only selects such plasmids but also aids in their persistence and dissemination. Here, the hybrid plasmids were a merger of IncFIB/IncHI1B (pNDM-MAR) backbones leading to convergence of AMR and virulence on a single plasmid. In India, *bla*_NDM_ is endemic and widespread among several bacteria especially *E. coli* and *K. pneumoniae* and this gene is often carried on the pNDM-MAR plasmid. Under pressure and for persistence, CR-HvKp can integrate segments of virulence plasmid into the endemic plasmid types to obtain the two-fold advantage of coding antimicrobial resistance and virulence. Similar plasmids, which were the result of the fusion between IncFIB/IncHI1B and IncFII_K_/IncFIB_K_ backbones, have been reported from other regions (Lam et al., 2019; Turton et al., 2019), highlighting the susceptibility of IncF and IncH plasmids to undergo recombination to form co-integrates in *K. pneumoniae*. Among these HvKp with mosaic plasmids, the mosaic structures were likely formed by the integration of the virulence region from the virulence plasmid into IncFIB and IncFII_K_ resistance plasmids (Lam et al., 2019; Tang et al., 2020).

Furthermore, we found that the MDR-HvKp clones carrying a hybrid virulence plasmid possessed a frameshift variant of *rmpA2* without *rmpA*. Mutations in *rmpA* and *rmpA2* led to a lack of hypermucoviscous phenotypes (Yu et al., 2015; Shankar et al., 2021b). The effect of inactivation of *rmpA2* and its stability on the hybrid plasmids carried by nosocomial clones of *K. pneumoniae* needs further investigation. In contrast, the hybrid plasmid in ST15 reported by Lam and colleagues carried only *rmpA* (Lam et al., 2019). The virulence plasmids described here also carried genes encoding heavy metal tolerance, such as tellurium and mercury. Similar hybrid plasmids, reported by Turton and colleagues, lacked the genes encoding for mercury tolerance (Turton et al., 2019). Given the community origin of HvKp, the co-occurrence of heavy-metal tolerance may provide an additional survival mechanism in harsh ecological niches found in the community and hospitals (Furlan et al., 2020).

The presence of CRISPR-Cas systems in MDR plasmids in *K. pneumoniae* has not been extensively studied. Recent reports of the type IV CRISPR-Cas system in *K. pneumoniae* mega plasmids/co-integrate plasmids suggest that these systems aid competition between plasmids (Kamruzzaman and Iredell, 2020; Newire et al., 2020). In the present study, the CRISPR-Cas system in the large co-integrate plasmid has acquired a spacer sequence identical to IncF-*traL*, which implies specific targeting for further gene invasion of plasmids (Kamruzzaman and Iredell, 2020). This might probably play a role in homologous recombination and integration of AMR and virulence determinants onto a single plasmid by preventing the entry of other plasmids such as IncF. The attainment of specific plasmid CRISPR spacers targeting different conjugative plasmids appears to be advantageous in *K. pneumoniae* to mitigate the fitness cost associated with carrying multiple AMR plasmids. Notably, the majority of the plasmids that carry plasmid-targeting spacers are co-integrate plasmids carrying IncFIB and IncHI1B replicons (Kamruzzaman and Iredell, 2020; Newire et al., 2020). This observation suggests that the plasmid-mediated CRISPR spacers not only target other plasmids but also may aid the formation of co-integrate/mega plasmids for improved stability and compatibility.

Globally, the prevalence of MDR-HvKp or CR-HvKp appears to be increasing (Siu et al., 2014; Gu et al., 2018; Liu et al., 2020). Given the large burden of MDR-HvKp and CR-HvKp infections in China, India, and Southeast Asia, these regions represent the most likely hotspot of MDR-virulence intersection and subsequent spread. Similarly, the spontaneous emergence of hybrid plasmids in these regions and their potential for clonal spread in healthcare settings represent a major focus of nosocomial outbreaks and their containment. If the incidence of the convergent clones with fusion plasmid continues, these pathotypes may replace the currently circulating CRKp clones (Liu et al., 2020). Continuous genome surveillance of MDR-HvKp would help in determining this niche shift from community to hospital. In China, ST11 was known to carry complete pLVPK with capsule-type K64 (clade1) while ST11 with K47 carried a shorter virulence plasmid with only *rmpA2* and aerobactin (clade3) (Dong et al., 2018; Liu et al., 2020; Zhang et al., 2020). These MDR-HvKp infections were predominantly associated with nosocomial infections, and the sub-lineages among ST11 with different capsule types were identified only through genomic studies. Although MDR-HvKp belonged to ST11 in the study described by Dong and colleagues, there are several differences concerning the virulence profile of the two clades which comprised MDR-HvKp which can only be identified through genome surveillance (Dong et al., 2018). There can also be a selection of one of the clades of MDR-HvKp ST11 over time which has a better fitness, thereby eliminating the other two clades.

## Conclusion

India has exceptionally high rates of AMR infections as stated by the annual report of the Antimicrobial Resistance Research and Surveillance Network during the year 2020 by the Indian Council of Medical Research (https://main.icmr.nic.in/sites/default/files/guidelines/AMRSN_annual_report_2020.pdf; accessed on November 15, 2021). The further generation of HvKp carrying carbapenemases on a virulence plasmid would be a potential catastrophe. The acquisition of AMR genes on the chromosome creates the further possibility of increased baseline resistance among the *K. pneumoniae* isolates. It is apparent that MDR-HvKp is no longer confined to selected clones and the containment of such isolates with the mosaic plasmid is very challenging. The presence of AMR and virulence among diverse *Klebsiella* clones present a global threat to the rapid spread of these emerging superbugs.

## Data Availability Statement

The datasets presented in this study can be found in GenBank under Bioproject ID PRJNA613369. The accession numbers of the genomes are mentioned in **Tables 2** and **3**.

## Ethics Statement

The study was approved by the Institutional Review Board of Christian Medical College, Vellore, India, with minute number 9616 (01/09/2015).

## Author Contributions

CS: conceptualization, analysis, manuscript writing, and revising. KV: methodology, bioinformatics, manuscript writing. JJ: analysis, manuscript writing and revising. SB: manuscript correction and supervision. BI: resource. AN: methodology, data curation. DS: methodology. BG: resource. BV: conceptualization, manuscript revision. and supervision. All authors contributed to the article and approved the submitted version.

## Funding

The study has been funded by the Indian Council of Medical Research, New Delhi, India (ref. no: AMR/Adoc/232/2020-ECD-II).

## Conflict of Interest

The authors declare that the research was conducted in the absence of any commercial or financial relationships that could be construed as a potential conflict of interest.

## Publisher’s Note

All claims expressed in this article are solely those of the authors and do not necessarily represent those of their affiliated organizations, or those of the publisher, the editors and the reviewers. Any product that may be evaluated in this article, or claim that may be made by its manufacturer, is not guaranteed or endorsed by the publisher.

## Abbreviations

ST, sequence type; MLST, multi-locus sequence typing; MDR, multidrug-resistant; AMR, antimicrobial resistance; HvKp, hypervirulent *Klebsiella pneumoniae*; CRKP, carbapenem-resistant *Klebsiella pneumoniae*; CR-HvKp, carbapenem-resistant hypervirulent *Klebsiella pneumoniae*; CG, clonal group; ARG, antimicrobial resistance genes; pLVPK, large virulence plasmid of *Klebsiella pneumoniae*; CRISPR, clustered regularly interspaced short palindromic repeats; XDR, extensively drug-resistant; SNP, single-nucleotide polymorphism; AST, antimicrobial susceptibility testing; OD, optical density.
