# Supplementary material for: Hybrid Plasmids Encoding Antimicrobial Resistance and Virulence Traits Among Hypervirulent Klebsiella pneumoniae ST2096 in India
Source: Front Cell Infect Microbiol. 2022 Apr 27;12:875116. doi: 10.3389/fcimb.2022.875116 (PMC9094440; doi:10.3389/fcimb.2022.875116)
Supplement: Supplementary file 1 [file DataSheet_1.pdf]

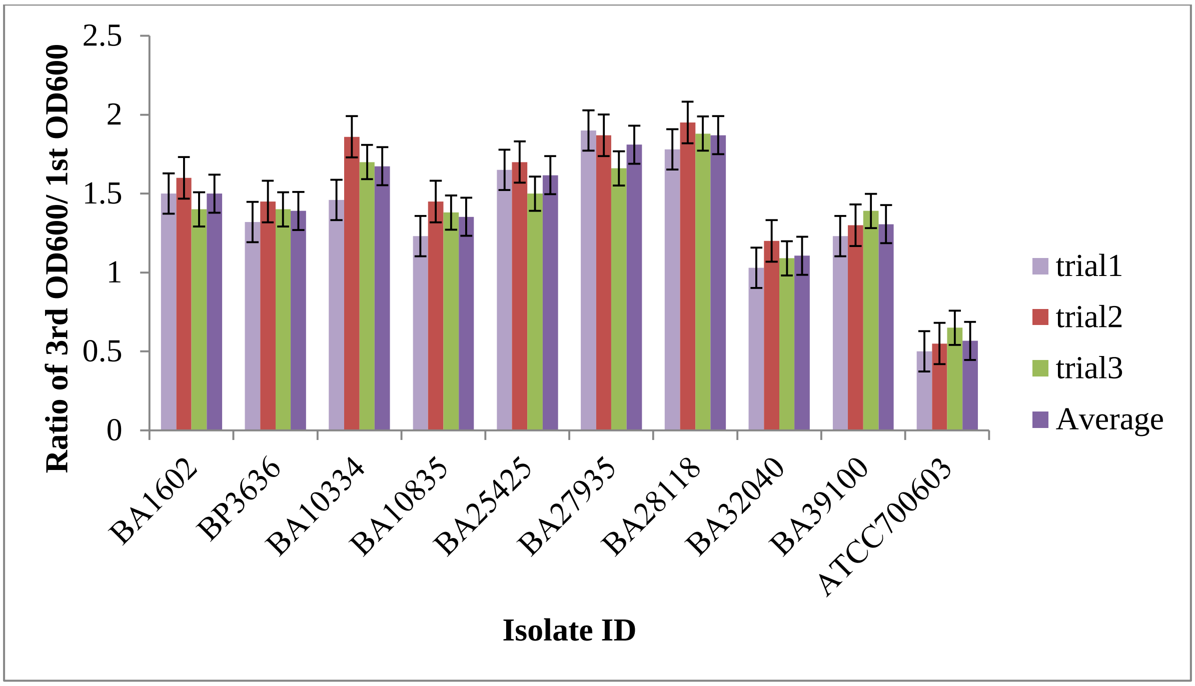

Figure S1: Results of mucoviscocity assay for the ST2096 hypervirulent *Klebsiella pneumoniae*

The OD600 was measured for all the isolates and compared against the OD600 of *Klebsiella quasipneumoniae* ATCC700603 as the reference since it lacks the virulence genes.

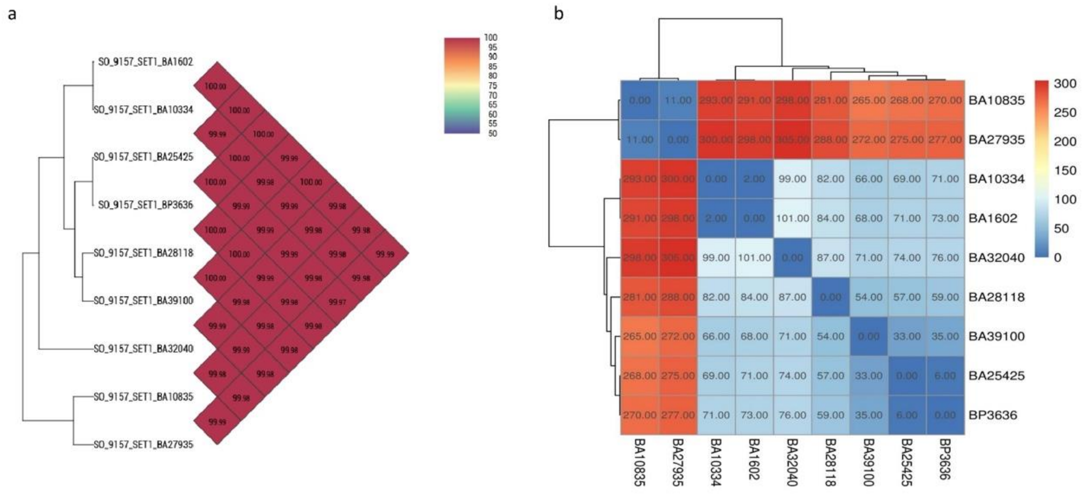

Figure S2: Average nucleotide index (ANI) of nine ST2096 MDR hypervirulent *K. pneumoniae*.

A)Heatmap generated from orthoANI values calculated from the OAT software. The nine study isolates formed two clusters of strains with isolates BA10835 and BA27935 being distinct from the remaining seven. **b)** Pairwise SNP distance table for all nine sequenced isolates calculated using SNP-dists v 0.6.3 (https://github.com/tseemann/snp-dists). Pairwise SNP difference between the nine study isolates identified two possible clusters with isolates BA10835 and BA27935 being >260 SNP distant from the rest.

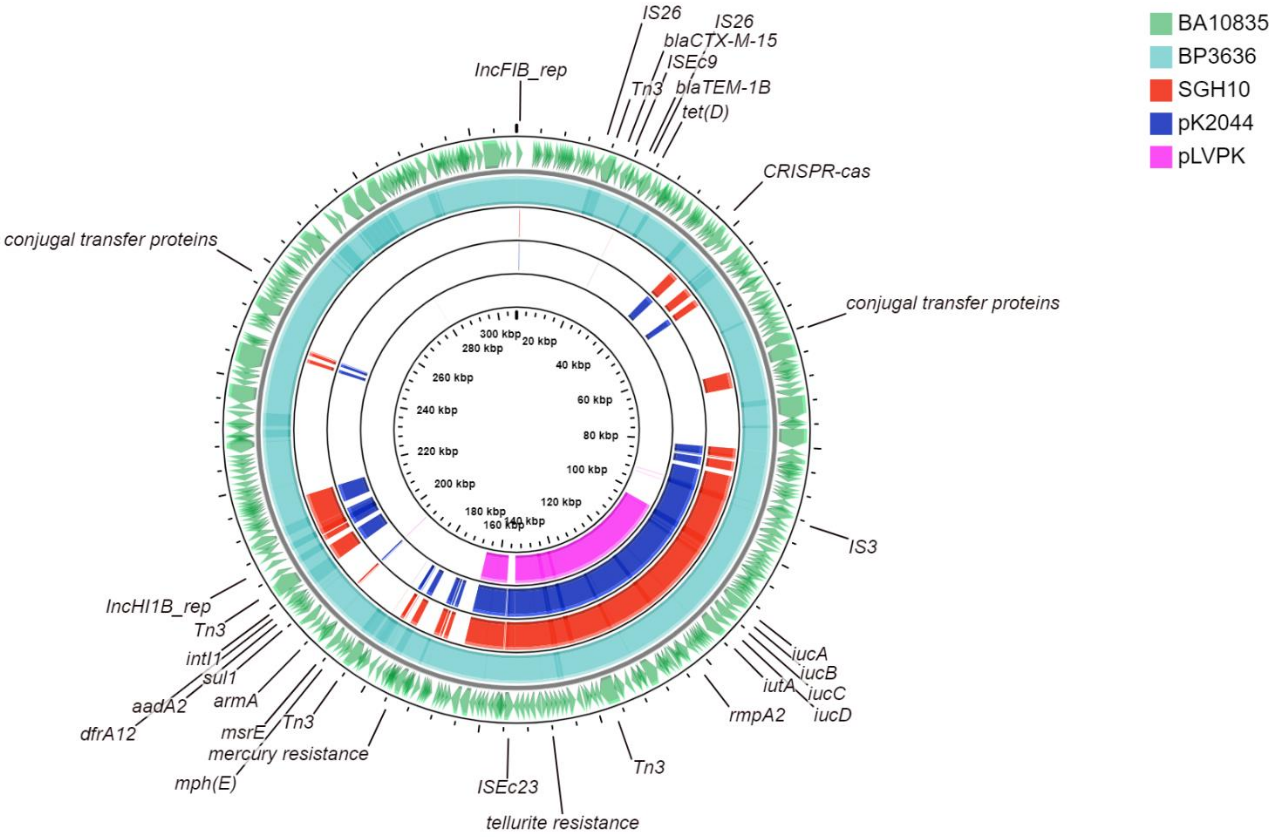

Figure S3: Circular view of comparison of the mosaic virulence plasmids from ST2096 with virulence plasmids of hypervirulent type strains.

Outside to inside rings: BA10835 (ST2096), BP3636 (ST2096), SGH10 (ST23, NZ_CP025081.1, 231Kbp) pK2044 (ST23, NC_006625.1, 224Kbp) and pLVPK (ST23, AY378100 , 68Kbp). Apart from the regions carrying the virulence genes, the mosaic plasmids vary from the typical virulence plasmids which are smaller in size and lack the IncFIB replicon. The regions carrying antimicrobial resistance genes and mercury resistance are absent in the virulence plasmids of the hypervirulent type strains.

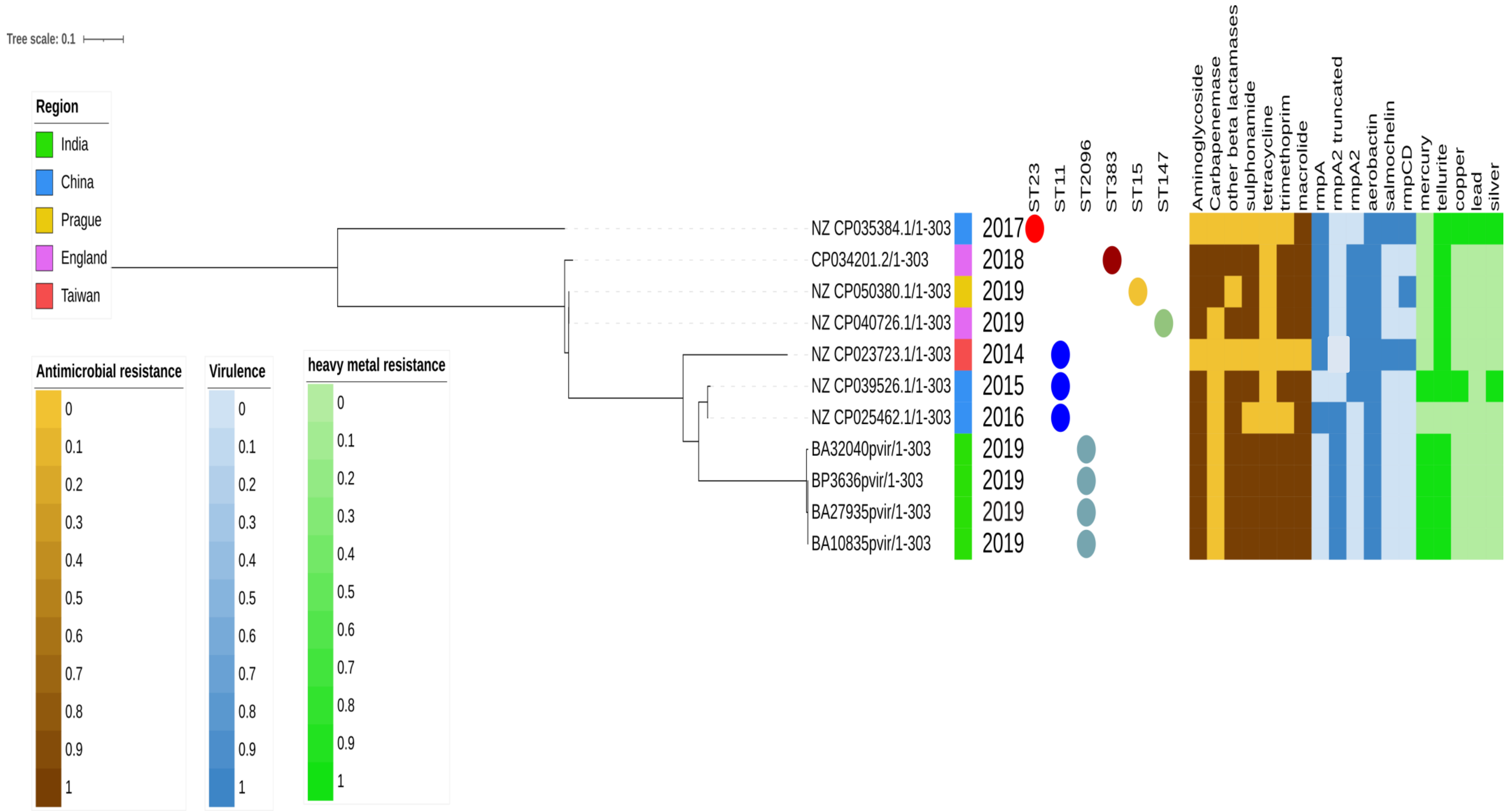

Figure S4: Phylogenetic tree of the IncHI1B-InFIB(pNDM-MAR) complete plasmids from global collection using CSI phylogeny

Table1: Characteristics of IncHI1B (pNDM-MAR)-InFIB (pNDM-MAR) mosaic plasmids from carbapenem resistant hypervirulent *Klebsiella pneumoniae*

**Accession number**	**CP053766[#]**	**CP053772[#]**	**CP034201**	**NZ_CP023723**	**NZ_CP025462**	**NZ_CP035384**	**NZ_CP039526**	**NZ_CP040726**	**NZ_CP050380**
**Plasmid name**	p10835_hyb	p3636_hyb	pKpvST383L	TVGHCRE225 plasmid unnamed1	p44-1	pAP855	pHB25-1-vir	pKpvST147B_virulence	p51015_NDM_1
**Size**	307718 bp	317680 bp	372826 bp	297984 bp	261706 bp	357837 bp	396373 bp	339117 bp	353810 bp
**Mobilization**	yes	yes	yes	yes	no	no	yes	no	yes
**AMR genes**	*aadA2, armA,* $bla_{\text{TEM-1B}}$, $bla_{\text{CTX-M-15}}$, *mphE, msrE, sul1, tetD, dfrA12*	*aac(6')-Ib-cr, aadA2, armA,* $bla_{\text{TEM-1A}}$, $bla_{\text{CTX-M-15}}$, $bla_{\text{OXA-1}}$, *msrE, mphE, sul1, tetD, dfrA12, dfrA14*	*APH(3')-VI, APH(3')-Ia, aadA1, armA, aac(6')-Ib, aac(6')-Ib-cr,* $bla_{\text{CTX-M-15}}$, $bla_{\text{OXA-9}}$, $bla_{\text{TEM-1}}$, $bla_{\text{NDM-5}}$, *qnrS1, mphA, mphE, msrE, sul1, sul2, dfrA5*	none	*aac(3)-IId, mphA,* $bla_{\text{CTX-M-15}}$, $bla_{\text{TEM-1B}}$	none	*aac3_IId, armA,aadA2, ant3pp_Ia,* $bla_{\text{TEM-1}}$, $bla_{\text{SHV-11}}$, $bla_{\text{DHA-1}}$, *qnrB, msrE, mphE, qnrB4,sul1, dfrA12, catA1*	*armA,* $bla_{\text{CTX-M-15}}$, *msrE, mphA, mphE, dfrA5, sul1, sul2*	*armA, aph3p_la, APH(3')-VI,* $bla_{\text{NDM-1}}$, *qnrS, msrE, mphA, mphE, dfrA5, sul1, sul2*
**Virulence genes**	*iucABCD, iutA, rmpA2**	*iucABCD, iutA, rmpA2**	*iucABCD, iutA, rmpA, rmpA2*	*rmpA, rmpA2, rmpC, rmpD, iucABCD, iutA, iroBCDN*	*rmpA, rmpA2*, iucABCD, iutA*	*rmpA, rmpC, rmpD, iucABCD, iutA, iroBCDN*	*rmpA2, iucABCD, iutA*	*rmpA, rmpA2, iucBCD, iutA*	*rmpA, rmpC, rmpD, iucABCD, iutA*
**Heavy metal resistance genes**	*merACDEPRT, terBDEWXZ*	*merACDEPRT, terBDEWXZ*	*terABCDEWXYZ*	*terABCDEWXYZ*	none	*pbrBCR, pcoABCDERS, silABCEFGPR, terABCDEWXYZ*	*merCPRT, pcoABCDER, silABCEFGPRS, terABCDEWXYZ*	*terABCDEWXYZ*	*terABCDEWXYZ*
**CRISPR-cas**	I-E	I-E	I-E	I-E	I-E	I-E	I-E	I-E	I-E
**Region and year of isolation**	India, 2019	India, 2019	England, 2018	Taiwan, 2014	Shanghai, China, 2016	Shanghai, China, 2017	China, 2015	England, 2019	Prague, 2019
**MLST of the isolate**	ST2096	ST2096	ST383	ST11	ST11	ST23	ST11	ST147	ST15
**Reference**	This study	This study	Turton et al., 2019	Huang et al., 2018	Newire et al., 2020	Tian et al., 2021	Xie et al., 2020	Turton et al., 2019	Chudejova et al., 2021

rmpA2 is frameshifted; [#]Isolates from this study

References:

- Chudejova, K., Kraftova, L., Mattioni Marchetti, V., Hrabak, J., Papagiannitsis, C.C. and Bitar, I., 2021. Genetic plurality of OXA/NDM-encoding features characterized from Enterobacterales recovered from Czech hospitals. *Frontiers in microbiology*, p.178.
- Huang, Y.H., Chou, S.H., Liang, S.W., Ni, C.E., Lin, Y.T., Huang, Y.W. and Yang, T.C., 2018. Emergence of an XDR and carbapenemase-producing hypervirulent *Klebsiella pneumoniae* strain in Taiwan. Journal of Antimicrobial Chemotherapy, 73(8), pp.2039-2046.

- Newire, E., Aydin, A., Juma, S., Enne, V.I. and Roberts, A.P., 2020. Identification of a Type IV-A CRISPR-Cas system located exclusively on IncHI1B/IncFIB plasmids in Enterobacteriaceae. *Frontiers in microbiology*, p.1937.
- Tian, D., Wang, M., Zhou, Y., Hu, D., Ou, H.Y. and Jiang, X., 2021. Genetic diversity and evolution of the virulence plasmids encoding aerobactin and salmochelin in *Klebsiella pneumoniae. Virulence*, *12*(1), pp.1323-1333.
- Turton, J., Davies, F., Turton, J., Perry, C., Payne, Z. and Pike, R., 2019. Hybrid resistance and virulence plasmids in “high-risk” clones of *Klebsiella pneumoniae*, including those carrying bla_{NDM-5}. *Microorganisms*, *7*(9), p.326.

- Xie, M., Chen, K., Ye, L., Yang, X., Xu, Q., Yang, C., Dong, N., Chan, E.W.C., Sun, Q., Shu, L. and Gu, D., 2020. Conjugation of virulence plasmid in clinical *Klebsiella pneumoniae* strains through formation of a fusion plasmid. *Advanced Biosystems*, *4*(4), p.1900239.
